# Health professionals’ perceptions of the barriers and facilitators to providing smoking cessation advice to women in pregnancy and during the post-partum period: a systematic review of qualitative research

**DOI:** 10.1186/s12889-016-2961-9

**Published:** 2016-03-31

**Authors:** Kate Flemming, Hilary Graham, Dorothy McCaughan, Kathryn Angus, Lesley Sinclair, Linda Bauld

**Affiliations:** Department of Health Sciences, University of York, York, YO10 5DD UK; Institute for Social Marketing and UK Centre for Tobacco and Alcohol Studies, University of Stirling, Stirling, FK9 4LA UK

**Keywords:** Pregnancy, Smoking, Health professionals, Qualitative research, Meta-ethnography, Systematic review

## Abstract

**Background:**

Reducing smoking in pregnancy is a policy priority in many countries and as a result there has been a rise in the development of services to help pregnant women to quit. A wide range of professionals are involved in providing these services, with midwives playing a particularly pivotal role. Understanding professionals’ experiences of providing smoking cessation support in pregnancy can help to inform the design of interventions as well as to improve routine care.

**Methods:**

A synthesis of qualitative research of health professionals’ perceptions of the barriers and facilitators to providing smoking cessation advice to women in pregnancy and the post-partum period was conducted using meta-ethnography. Searches were undertaken from 1990 to January 2015 using terms for maternity health professionals and smoking cessation advisors, pregnancy, post-partum, smoking, and qualitative in seven electronic databases. The review was reported in accordance with the ‘Enhancing transparency in reporting the synthesis of qualitative research’ (ENTREQ) statement.

**Results:**

Eight studies reported in nine papers were included, reporting on the views of 190 health professionals/key informants, including 85 midwives and health visitors. The synthesis identified that both the professional role of participants and the organisational context in which they worked could act as either barriers or facilitators to an individual’s ability to provide smoking cessation support to pregnant or post-partum women. Underpinning these factors was an acknowledgment that the association between maternal smoking and social disadvantage was a considerable barrier to addressing and supporting smoking cessation

**Conclusions:**

The review identifies a role for professional education, both pre-qualification and in continuing professional development that will enable individuals to provide smoking cessation support to pregnant women. Key to the success of this education is recognising the centrality of the professional-client/patient relationship in any interaction. The review also highlights a widespread professional perception of the barriers associated with helping women give up smoking in pregnancy, particularly for those in disadvantaged circumstances. Improving the quality and accessibility of evidence on effective healthcare interventions, including evidence on ‘what works’ to support smoking cessation in disadvantaged groups, should therefore be a priority.

**PROSPERO 2013:**

CRD42013004170.

**Electronic supplementary material:**

The online version of this article (doi:10.1186/s12889-016-2961-9) contains supplementary material, which is available to authorized users.

## Background

Reducing smoking in pregnancy is a policy priority in many countries [1]. In the UK, for example, targets to reduce smoking in pregnancy have been supported by investment in tailored smoking cessation services to provide support to women who find it difficult to stop [2]. However, smoking rates remain high particularly for women in disadvantaged circumstances, groups who also tend to be less well-served by maternity care services [3–6]. For example, in England 12 % of pregnant women are recorded as smoking at the time of delivery, which translates into over 83,000 infants born to smoking mothers each year. Pregnant women from unskilled occupation groups are five times more likely to smoke than professionals, and teenagers are six times more likely to smoke than older mothers in England [7].

Those providing these services play a vital role in supporting healthy lifestyles in pregnancy [8, 9], in particular the opportunity to counsel both behaviour change at a time when individuals are receptive to teaching [10]. A wide range of professionals are involved, including obstetricians, family doctors, nurses and pharmacists. In a number of countries, midwives play a particularly pivotal role including in raising the issue of smoking cessation, offering behavioural support and referring to specialist services [11, 12]. However, midwives and other healthcare providers can lack knowledge and confidence for this role, and may also struggle to find adequate time during busy antenatal appointments [13]. Understanding their experiences of providing smoking cessation support in pregnancy can help to inform the design of interventions as well as to improve routine care.

Qualitative studies are often the research design of choice for capturing subjective perceptions and experiences, and can offer unique insights for tobacco control policy and practice. For example, qualitative studies have contributed to understanding how to introduce and enforce smokefree policies and point of sale display regulations [14–17]. However, systematic reviews of qualitative studies are rare. With respect to women’s experiences of smoking and smoking cessation in pregnancy and post-partum, systematic reviews of qualitative studies are now beginning to fill this gap [18–20]. Yet, despite their pivotal role, there have been no systematic reviews of qualitative studies of healthcare providers’ perceptions and experiences of providing advice and support around smoking cessation in and after pregnancy.

This review aimed to explore the barriers and facilitators to supporting smoking cessation in pregnancy and after birth from the perspective of health professionals. The paper presents a synthesis of qualitative studies conducted in high-income countries that collected evidence on health professionals’ perceptions and experiences.

## Methods

### Design

A synthesis of qualitative studies exploring health professionals’ perceptions and experiences of the barriers and facilitators to supporting smoking cessation during pregnancy and post-partum was conducted using meta-ethnography [21]. Meta-ethnography is an interpretative approach to research synthesis which enables conceptual translation between different types of qualitative research [22].

### Search methods

**Fig. 1:**
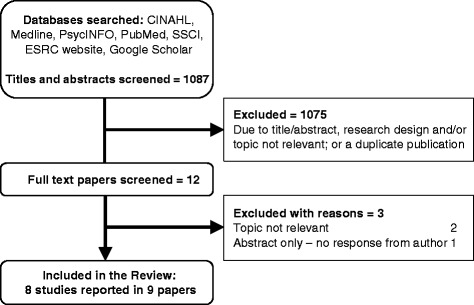
Flow chart of study inclusion and exclusion

We searched for published and unpublished studies from 1990 to January 2015 (Fig. 1). Terms for smoking cessation, pregnancy, post-partum, maternity health professionals and smoking cessation advisors, were developed for searches of electronic databases (CINAHL, MEDLINE, PsycINFO, Social Sciences Citation Index (SSCI), Economic and Social Research Council (ESRC) website, and a specific ‘ahead of print’ search in PubMed and Google Scholar) on 25-28^th^ February 2014, together with citation searching and consultation with the wider project team. Detail of the search strategy is provided in Additional file 1.

Papers from 1990 were selected for inclusion if they (a) were published in English and reported on health professionals’ experiences of supporting smoking cessation during pregnancy and post-partum, (b) used a qualitative research method and (c) were conducted in a high income country (as defined by the World Bank [23]) where, as in the UK, cigarette smoking is associated with social disadvantage.

### Data extraction and quality appraisal

Relevant data were extracted from papers (aim, type and number of participants, methodology used, methods of data collection, analysis, and results). Data were extracted by one reviewer (KF) and checked by another (DM). Papers were appraised for quality using an established checklist for qualitative research [24] by two reviewers (KF, DM), with disagreements in scoring resolved by consensus. The checklist included assessment of both the conduct and reporting of each paper against a pre-determined set of descriptors. Quality scores ranged from 19-29 (Table 2). The lower scoring papers tended to lack depth of description regarding research methods, issues around ethics and the reporting of findings. There was no a priori quality threshold for excluding papers; assessment was undertaken to ensure transparency in the process.

### Method of synthesis

Meta-ethnography has four iterative phases (Table 1). For Phase 1, three reviewers (KF, HG, DM) read all papers in depth. Phase 2 involved line-by-line coding of data (participant accounts and authors’ interpretations) in each paper (KF) relating to health professionals’ perceptions and experience of smoking cessation during pregnancy and post-partum using ATLAS.ti Software [25].

**Table 1:** Phases of meta-ethnography (adapted from Noblit and Hare [21]) [22]

Phase of meta-ethnography	Processes involved
Phase 1 Reading the studies	Developing an understanding of each study’s context and findings.
Phase 2 Determining how the studies are related	Comparing contexts and findings across and between studies.
Phase 3 Translating the studies into one another	Mapping similarities and differences in findings and translating them into one another; the translations represent a reduced account of all studies. (First level of synthesis)
Phase 4 Synthesising translations	Identifying translations that encompass each other and can be further synthesised; expressed as ‘lines of argument’. (Second level of synthesis)

The codes were compared and grouped by the reviewers (KF, DM, HG) into broad areas of similarity through reciprocal translation analysis (RTA) (Phase 3) to generate a reduced set of codes (translations) about barriers and facilitors that health professionals perceived related to women’s smoking cessation. Phase 4 focused on these translations; the reviewers (KF, DM, HG) examined and compared them to identify ‘lines of argument’. These capture health professionals’ perceptions and experience of the barriers and facilitators they face when providing smoking cessation support.

## Results

Of 1087 potentially-relevant papers, 1075 were excluded. Eight studies reported in nine papers were included in the review (Fig. 1, Table 2).

**Table 2:** Included papers (*n* = 9) grouped by study (*n* = 8) (*denotes the related papers)

Source Paper (*n* = 9)	Country setting	Aim	Participants	Methodology	Indicative finding	Quality Score (out of 32)
Abrahamsson A, Springett J, Karlsson L et al (2005) [26]	Sweden	To describe the qualitatively different ways in which midwives make sense of how to approach women smokers	Midwives (*n* = 24) purposively sampled, who had been offered training in person-centred methods. Experience 2-24 years	Phenomenology	Midwives used different approaches to address smoking with pregnant women. Four different ‘story types’ were identified: avoiding, informing, friend-making and co-operating.	25
Aquilino ML, Goody CM, Lowe JB (2003) [31]	USA	To examine the perspectives of Women, Infants & Children (WIC) clinic providers on offering smoking cessation interventions for pregnant women	Four focus groups (*n* = 25) consisting of WIC nurses (*n* = 14), dieticians (*n* = 9) and social workers (*n* = 2). Three participants revealed that they smoked	Data collected via focus groups and analysis was undertaken using ‘code mapping’	Factors affecting WIC staff’s provision of smoking cessation information were: time, competing priorities, staff approaches to clients, staff training, nature of educational materials and client concerns.	24
Borland T, Babayan A, Irfan S et al (2013) [32]	Canada	To explore how Ontario’s cessation policy, programming and practice encourage or discourage the provision and uptake of support by women	Key informants (*n* = 31) from provincial organisations that offer cessation, maternal and/or child health support to women across Ontario	Data collected by semi-structured in-depth interviews. Data were analysed using thematic interpretive analysis	Key barriers to providing cessation support included: the absence of a provincial cessation strategy and funding; capacity issues; lack of a programme that was woman-centred, included the social determinants of health and the needs of specific groups; inconsistent practice; geographical factors.	27
Bull (2007) [27]	UK	To explore the role of midwives and health visitors in the prevention of smoking during pregnancy and early parenthood	Health visitors (*n* = 16) and midwives (*n* = 7)	Data were collected via two focus groups and analysed using qualitative content analysis	Midwives and health visitors are willing to accept professional responsibility for smoking cessation work with their patients. They perceive their role as being limited by the socio-economic circumstances of their clients and recognise that they additionally must be ‘ready to change’.	20
Ebert M, Freeman L, Fahy K et al (2009) [28]	Australia	To determine how midwives interact with women who smoke in pregnancy in relation to the women’s health and well being	Community midwives (*n* = 7) each with a minimum of 6 years’ experience (research initially wanted to looked at midwife/woman dyads but no women were recruited).	Interpretive interactionism design and analysis. Data collected through two individual interviews with each midwife.	Whilst midwives acknowledge they need to engage in woman centred dialogue during smoking cessation interactions, more commonly the engagement was limited to predictable, planned and computer prompted interactions.	19
Herberts C & Sykes C (2011) [29]	UK	To identify and juxtapose midwives’ perceptions of providing stop-smoking advice and pregnant smokers’ perceptions of stop-smoking services	Midwives (*n* = 15) recruited from 2 acute trusts in the borough of Camden (19^th^ most deprived borough in England)	Three focus groups centred on the key question ‘How do you feel about talking to pregnant women about smoking cessation?’ Data analysed using constructs of grounded theory	Midwives identified both barriers and facilitators to providing stop-smoking advice. Barriers included: fear of being seen to judge women, putting pressure on women, threatening the professional relationship, lack of education to provide support, insufficient time. Facilitators included: being more experienced, being an ex-smoker, having sufficient levels of relevant knowledge, time, a good relationship with the woman and continuity of care.	29
* Herzig K, Danley D, Jackson R et al (2006) [33]	USA	To explore prenatal providers’ methods for identifying and counselling pregnant women to reduce or stop smoking, alcohol use, illicit drug use and the risk of domestic violence	Obstetricians/gynaecologists (*n* = 40), nurse midwives (*n* = 5), nurse practitioners (*n* = 3), registered nurse, working in HMO (*n* = 1), private practice, community health clinics, hospitals and academic centres	Six focus groups with 6-11 participants in each, questioning led by an open-ended question guide. Data were analysed using a subjective, interpretive ‘editing style’ of analysis	Participants talk of specific risk prevention methods used with pregnant women who smoke (amongst the 4 risk factors studied), citing a patient centred collaborative style as particularly helpful. Harm reduction strategies rather than abstinence were recommended, along with incorporating the wider family.	26
* Herzig K, Huynh D, Gilbert et al (2006) [34]	USA	To explore prenatal providers’ methods for addressing four behavioural risks in their pregnant patients: alcohol, drug use, smoking and domestic violence	Obstetricians/gynaecologists (*n* = 40), nurse midwives (*n* = 5), nurse practitioners (*n* = 3), registered nurse, working in HMO (*n* = 1), private practice, community health clinics, hospitals and academic centres	Six focus groups with 6-11 participants in each, questioning led by an open-ended question guide. Data were analysed using a subjective, interpretive ‘editing style’ of analysis	The study addresses each of the four behavioural risks. Smoking was seen as the ‘easiest’ risk to address, but its addictive quality proved challenging to overcome.	26
McLeod D, Benn C, Pullon S et al (2003) [30]	New Zealand	To explore the midwife’s role in providing education and support for changes in smoking behaviour during usual primary maternity care	Midwives (*n* = 16) with between 5-20+ years in practice, who had been part of a RCT of education and support for pregnant women who smoke. Midwives had either received smoking cessation training as part of the trial (*n* = 9), or had received no such training (*n* = 7)	Data were collected through individual interviews. Midwives additionally completed a postal questionnaire, asking about education, training, smoking status, and perception of barriers to delivering smoking cessation advice	Providing smoking cessation support was seen as part of the midwife’s role, but it was perceived as difficult to start conversations on the subject, to identify women who would be receptive and to support them. There was concern over the impact of providing cessation advice on their relationship with women.	25

The eight studies reported on the views of 190 health professionals/key informants. Five studies included midwives (*n* = 69) or health visitors (*n* = 16) only [26–30]. The remaining three studies focused on Women, Infants & Children (WIC) nurses, social workers and dieticians [31], key informants and child health support workers from provincial organisations [32] and obstetricians and gynaecologists, with a lesser focus on nurse midwives [33, 34]. Two studies [27, 29] were conducted in the UK (*n* = 22 midwives and 15 health visitors), and two in the USA [31, 33, 34]. The remaining four studies were conducted in Australia, Canada, New Zealand and Sweden. Across the different professional roles included in the review, health professionals and key informants were likely to care for women variously in: the ante-natal period; the ante-natal and post-natal period; the post-natal period. Commonly professionals did not clarify which group they were referring to when they spoke of their smoking cessation role.

The meta-ethnography identified two lines of argument running through health professionals’ accounts of their experiences of providing smoking cessation support to women in pregnancy and in the post-partum period: their professional role and the organisational context in which they worked. These lines of argument relate to two closely linked contexts central to health professionals’ interactions with women, each with the potential to facilitate and also act as a barrier to smoking cessation. These lines of argument are described below. Job titles are given where these are available; titles can vary between countries.

### Professional role

This line of argument highlighted aspects of the professional’s identity with the potential to facilitate support-giving around smoking cessation. Key aspects were: their approaches to smoking cessation, their professional role and skills, their relationship with the patient/client and their professional perceptions. These positive aspects were not however fixed and invariant; the balance could tip and become an ensuing barrier.

#### Experience-based facilitators to smoking cessation

Studies containing a mix of professionals, including midwives, specialist nurses, obstetricians and support workers, described a range of approaches that participants identified as helpful [26, 30, 31, 33]. These strategies had been learned both through their training and their experience of working with pregnant smokers.

Short interactions that briefly engaged with smoking cessation were favoured, with professionals promoting small positive steps to cutting down or quitting that helped women feel in control [26, 30, 31, 33].

*‘…it didn’t have to be a big issue, but I think you could still get your message across fairly succinctly just by bringing it up reasonably frequently, but just little jabby thoughts.’* Midwife [30]

*‘…I’ll say ‘Okay, all you have to do this month is just not smoke in the car.’ That will count for a percentage…and they’ll come back, and say ‘Okay, I only smoked in the car one time,’ and that’s okay.’* Obstetrician [33]

*‘If they say they’ve thought about giving up and that it’s hard now, then you have to say it’s good they’ve thought about it…I try to make the most of the positive things they’ve done.’* Midwife [26]

Such approaches could also include a focus on the unborn baby’s health, alongside encouraging other positive health behaviours that women regarded as incompatible with smoking, such as breastfeeding [26, 30].

*‘When you ask if they smoke, they sigh and say it’s not good, because they know the question’s coming. I explain and show the leaflet about how dangerous it is and that they must think about the baby.’* Midwife [26]

*‘I think sometimes focusing on that really positive thing -- breast feeding your baby -- allows messages about smoking to be drip fed in.’* Midwife [30]

Professionals saw women as responsible for their own behaviour change; placing the woman at the centre of her decision to quit was therefore important. To be effective, discussing smoking cessation required sensitivity and tact [27]. This required professionals to assess the woman’s motivation to quit and develop approaches appropriate to her stage of change, skills which drew on their interpersonal and counselling skills [26, 30–33]. It was acknowledged that change may be slow but, as professionals, they may be investing in future cessation [33].

*‘We try not to be judgmental and I try not to pass judgment, but I just tell them that whatever you do that baby’s getting, so if you're getting your little smoke on, they’re getting their little smoke on, too.’* [31]

*‘It makes a difference to talk to the women. It may not be our joy to see any change, but change may happen another time. In the meantime I want to keep her and her foetus as safe as possible.’* Nurse Midwife [33]

Helping women to understand how smoking affected their baby provided another approach, for example through easy-to-read, straightforward graphical information [26, 31].

*‘…I say that the baby becomes smaller due to the lack of nourishment, that it has a smaller refrigerator, thinner arteries. If they still don’t get it I show them a pretty horrible picture.’* Midwife [26]

*‘Sometimes I even draw a picture, very crudely, of a red blood cell and carbon monoxide and oxygen, how it [smoking] knocks off the oxygen so the body has to make more, and they seem to understand that.’* [31]

The involvement of partners was also discussed [27, 30, 32, 33]. It was recognised that opportunities to work with partners were limited and they commonly knew little about the risks of smoking in pregnancy or around second or third hand smoke. Therefore the need to ‘grab every opportunity to get the point across’ was paramount [27].

*‘No way to get to them, it hasn’t actually been talked about. Like the woman I see right now, I mean her partner smokes like a chimney and it is not helping her at all… but I never see him.’* Health Visitor [27]

Generally and where possible, it was seen as advantageous to include partners in smoking cessation advice and education. Partner engagement and support for the woman’s cessation, either through joint quitting or cutting down, was regarded as a key determinant of success [30, 32, 33].

*‘I think one of the patient’s real barriers to success is the spouse or somebody living with them who is still smoking, so I’ll give out prescriptions for the patch to husbands.’* Obstetrician [33]

#### Health professionals’ roles and skills

Striving to support smoking cessation was recognised to be a key part of the professional’s role [26, 30].

*‘It’s part and parcel of the job. No, it’s an intrinsic part of it…I mean pregnancy and childbirth is such a holistic period that you can’t compartmentalise and just deal with one aspect.’* Midwife [30]

It was acknowledged that this role required up-to-date, relevant knowledge and experience as well as supportive organisational structures [29]. With respect to knowledge and experience, the need to appreciate the context of maternal smoking was noted, including the role that smoking played in the lives of their patients, the importance of positive messaging and practicing in an empathetic manner [26].

Professionals noted the importance of education and training – and the lack of confidence that skills deficits could induce producing a barrier to providing support to women. Skills gaps included how to open up the issue of smoking cessation, as well as how to follow up these initial discussions [26–31]. Frequently, professionals felt they lacked the knowledge and skills to deliver information in a way that would be well-received by women, with a resulting unease about ‘getting it wrong’ [27–32].

*‘We haven’t been trained about how to do it, so you get it wrong don’t you?’* Health Visitor [27]

*‘I could use more information. There’s new stuff every day that relates to smoking, so I know there’s new and up-to-date stuff that we probably don’t know about.’* [31]

*‘Sometimes you don’t know what to do. You don’t want to scratch the surface if you can’t follow it up.’* Midwife [26]

Compounding these concerns were organisational constraints and a sense that, with their client, supporting behaviour was challenging and available interventions were ineffective [27, 30].

*‘Not enough time and not a special interest of mine since they don’t stop smoking.’* Midwife [27]

*‘We have too much to do with booking and like everyone else says it takes too much time and I don’t know what works!’* Midwife [27]

An additional concern voiced by UK health visitors and midwives [27] and by province-wide key informants in Canada [32] concerned Nicotine Replacement Therapy (NRT) in pregnancy. Participants in the UK study spoke of inconsistent advice and an absence of clinical leadership, alongside uncertainty over its licensing for use in pregnancy and a lack of guidance over its prescribing [27]. Reservations over the use of NRT were expressed.

*‘Well the women don’t like using it so compliance is an issue. Are we all pinning our hopes on something that doesn’t do the trick?’* Midwife [27]

*‘…if there [was a] dictum or policy that comes down that says, ‘We fully support the use by prenatal women of nicotine replacement under recommendation from pharmacists,' that would go a long way to providing additional support and services.’* Key informant [32]

#### The relationship with the pregnant woman

Study participants made clear that the relationship with the pregnant woman was central to meeting their professional responsibilities to her and her baby. A positive relationship provided the platform and helped to facilitate smoking cessation, but it could take time to develop, particularly where continuity of care was limited. In circumstances where relationships were more difficult to form, it was acknowledged that the absence of a relationship, or one that was less than positive could act as a barrier to providing support.

Many professionals talked of a tension between maintaining a positive relationship and addressing the issue of smoking.

*‘…you have a special relationship with the woman because you meet so many times. You want to be professional and… create a sense of security… You don’t want to be known as a nagging old cow.’* Midwife [26]

This tension was managed in a range of ways. A commonly-reported response was to approach conversations about smoking cautiously, for example by ensuring that information was not offered unless the woman had asked for it and could see the use of it [26, 27, 29]. Some professionals were concerned that even asking about smoking status could adversely affect the relationship [30], as could repeatedly raising the subject at subsequent appointments [27, 29, 30].

*‘If people sort of give you the impression from the beginning that they are not interested in changing their smoking habits then I think it could be detrimental to our relationship if I was to bring it up every time.’* Midwife [30]

*‘I do talk about smoking cessation with them, reinforcing what they’ve already heard, sometimes…they’re receptive to it and other times, it’s like they have heard it from everyone that day and it’s almost like you can see the door closing.’* [31]

This widespread caution arose from previous experiences of the negative effects of discussing smoking and smoking cessation [26, 27, 29]. Raising these issues could therefore be risky, potentially alienating the woman from other essential pregnancy-related support and advice, particularly for vulnerable women [26, 27]. Some professionals acknowledged that their concerns meant that they avoided confronting a significant health risk – and thus failed to fulfil their professional responsibilities to mother and baby [26].

*‘Yes, maybe I should get to grips with the smoking because it isn’t good for the baby or the mother. I feel bad about not doing it, but… I’ve chosen not to because I want to keep the mother’s trust.’* Midwife [26]

However, other professionals reported that they did not avoid conversations about smoking, recognising that, however difficult, there was a professional requirement to give information and advice. Women were assumed to know the risks of smoking in pregnancy and would therefore be primed to discuss smoking [26]. They acknowledged that guilt and defensiveness were to be expected, although this depended in part on the way information was presented [26, 30, 31].

*‘I really think you have to be frank in what you say. Of course you make them feel guilty. You do it automatically in a way.’* Midwife [26]

*‘It’s one of those topics that’s hard to talk about…they think you’re lecturing them on something bad and…[they] immediately get defensive.’* [31]

While concerns about negative effects on the professional-woman relationship predominated, there were also examples of positive experiences. These typically occurred where professionals were confident that women wanted to make changes to their smoking and smoking cessation support was welcomed [30].

*‘Those that were interested in trying to give up smoking were…quite appreciative that somebody was trying to take the time and effort to try and help them’* Midwife [30]

#### Appreciation of women’s lives and the context of their smoking

The studies contributing to this section described professional perceptions of why women smoke in pregnancy and why smoking cessation was challenging. Identifying and understanding these perceptions can help to identify facilitators and barriers to supporting smoking cessation that may otherwise remain hidden. Perceptions focused primarily around the place of an addictive behaviour in disadvantaged lives and in communities where smoking was the norm.

Women living in disadvantaged circumstances with many life stressors and demands were perceived as prioritising immediate needs over smoking cessation [27, 29–31]. In such contexts, smoking was seen as a source of support; a way of getting by day-to-day. It was acknowledged that pregnancy could be a difficult time for smoking cessation, although the professional responsibility to encourage it remained [30, 31].

*‘Sometimes it’s just not the right time. And they know, they know what they’re doing and um yeah, and some people are in such awful situations that it’s sort of like it’s their only bit of self-indulgence and yet…’* Midwife [30]

*‘Sometimes they have so many stressors in their life that they just don’t think they can give it (smoking) up, and that’s probably true.’* [31]

There was also a perception that smoking was a lifelong and habitual behaviour [27, 30, 34], unquestioned until a life event like pregnancy occurred [30]. The addictive nature of smoking, and the difficulties for women who wanted to attempt quitting, were also acknowledged [27, 34]:

*‘(Name) started at age six when she used to light cigarettes from the coal range for her mother who stayed in bed.’* [30]

In Bull’s study of midwives and health visitors, there was also recognition that smoking may be experienced as therapeutic, particularly for women whose mental health was poor, a dimension that added to the challenges of providing sensitive support for quitting [27].

Looking beyond the woman to her wider environment, professionals acknowledged that this could also be a barrier both to attempting to quit and to subsequent abstinence [30, 31]. Perceived barriers included the smoking habits of family and friends, with partners’ smoking habits seen as particularly influential.

*‘…he just carried on smoking in the house, in the lounge, and that girl really wanted him to smoke outside, but he was just the male bulshie, and I wasn’t going to cross him. I mean you can feel vibes.’* Midwife [30]

Professionals supporting women living complex and challenging lives talked about how they would promote harm reduction, advising women to cut down rather than quit. This was seen as a more feasible option: less stressful for the woman and less likely to make her feel ‘got at’ by professionals with repeated messages about cessation [30, 31, 33].

*‘I don’t recall that I ever saw many women who completely stopped [smoking]. ..We always said that any reduction is an improvement and will help with the outcome of the baby…’* [31]

*‘I mostly encourage them to cut down I don’t think stopping is a good option for the majority of women. The odd one will stop but yeah. There’s confirmed smokers who will never stop.’* Midwife [30]

For those women who were successful in quitting during pregnancy, professionals expected that it may well be short term, undertaken for the sake of the baby [27, 30, 31].

*‘I looked at my own statistics and then rang my own women round, and asked them if they’d gone back to smoking when the baby was delivered and sadly the majority had.’* Midwife [30]

The study by Bull [27] noted scepticism about whether post-partum relapse was avoidable, along with recognition that professionals lacked knowledge about effective interventions to prevent it.

*‘I think it is very difficult… to give up for pregnancy is about giving up for the baby, and I don’t think there is any preparation or support about how to give up long term as a non-smoker afterwards.’* Health Visitor [27]

Alongside the emphasis on the challenges of women’s lives were insights into women’s risk perceptions and how these perceptions could make conversations about quitting difficult. Professionals noted that women struggled to fully understand the risks of smoking in pregnancy and relate these risks to her own pregnancy [29, 34]. Professionals also recognised that the risk behaviours to which they gave emphasis may not be the ones that women perceived as risky [34].

*‘I had one [patient] who was on methadone and also smoked… I said, “....You’re early in your first trimester. You can’t smoke…” she said, “What are you talking about, I can’t smoke?” She was expecting a conversation about the methadone.’* Obstetrician/Gynaecologist [34]

This section has addressed health professionals’ awareness as to how their role, their relationship with women and the difficult circumstances in which women live their lives, can, depending on context act as a facilitator or barrier to their approach and strategies to provide smoking cessation support.

### Organisational context

The impact of organisational contexts was evident in the line of argument centred on the professional role. These contexts also emerged as direct influence on both the facilitators for, and the barriers to, the provision of support for smoking cessation. Organisation was described at two levels: organisation of services and organisation of individual professional practice.

#### Organisation of services

Evidence on the impact of service configuration and delivery came predominantly from two studies [27, 32]. The first study interviewed individuals working for provincial organisations offering cessation support and maternal and child health care to women across Ontario, Canada [32]. The second was a UK-based study with a broader focus on the role of midwives and health visitors in smoking cessation in pregnancy and early parenthood [27].

The Canadian study highlighted two linked factors: the importance of explicit policies shared across organisations and adequate resources to deliver them. The study discussed the need for centralised cessation policies, practices and procedures focussed on working directly with pregnant and postpartum women who smoke; absence of such structures was perceived as a barrier to providing smoking cessation support. Additionally, developing systematic relationships between organisations, practitioners and experts working on smoking cessation was seen to facilitate shared learning, referral pathways and intervention development.

The barriers of weak polices and organisational frameworks were perceived to be compounded by lack of funding. Without continuity of funding, building a system of co-ordinated services, with trained professionals working with women during and after pregnancy, was seen to be impossible.

‘*We don’t have the resources, we don’t have the clinicians, we don’t have the tobacco replacement system…We don’t have any of those.’* Key informant [32]

Resources were also needed to address barriers to women being able to access support, for example, transport to clinics and the provision of childcare. Locally-based venues and home visits were seen as ways of improving women’s access to smoking cessation services. Secure funding would also enable the adaptation of programmes to meet the needs of particular groups, for example minority groups and adolescents.

The UK study focussed on different but complementary aspects [27]. One key insight related to the perceived framing of smoking cessation support in pregnancy as a clinical issue, delivered within healthcare settings, rather than a social issue, addressed in community settings. It was considered that, to facilitate smoking cessation to be integrated into smokers’ lives, it needed to be tackled in many contexts and forms of media, a perspective linking to the Canadian professionals’ perception that services should be available in the community.

*‘Different ways [are needed] other than the medical model of giving advice which clearly doesn’t work with this group of women…It is not seen within the social context of how they are living; just the health field.’* Health Visitor [27]

Health professionals considered that the social influences on smoking and a woman’s ability to quit are not acknowledged in wider government policies or in targets for smoking cessation, acting as a barrier to the effectiveness of these interventions. Post-partum relapse was similarly linked to the failure to address wider determinants and institute stronger anti-poverty policies.

*‘How can we be expected to change that [poverty-related smoking]! It is quite frightening when local Trusts are being performance monitored and you are held accountable to them when in fact the causes are way outside your control.’* Health Visitor [27]

Linked to the perception that smoking in pregnancy was a social issue, the midwives and health visitors in Bull’s study questioned whether smoking cessation advice was best delivered by health professionals [27]. For example, it was noted that former smokers, with an experience of tobacco dependence that many professionals lacked, could be employed as smoking cessation advisors, facilitating the effectiveness of this role. The idea of ex-smokers as role models and advisors was briefly mentioned in two other studies [29, 30].

*‘You should be training a lay person, like an ex-smoker, as they maybe more accepted for being there and showing concern. A mother herself maybe could help others to quit.’* Health Visitor [27]

*‘I tell them that I did it so they can jolly well do it too. Because I’ve smoked. That is actually quite a valuable tool.’* Midwife [30]

#### Organisation of individual practice

Health professionals described how the organisation of their individual practice could facilitate or hinder their ability to deliver smoking cessation advice to women.

For example, organisational requirements could determine when and how midwives discussed smoking [26, 28]. Organisational requirements to ask and record smoking status in prescribed ways were perceived as barriers to woman-centred communication. Midwives described how computerised prompts, with their closed questions such as ‘do you smoke?’ were the main trigger for initiating communication about smoking. While ensuring compliance with organisational procedures and meeting minimum professional duties, it inhibited more sensitive approaches to smoking status and smoking cessation.

*‘One of the questions in our booking-in database asked specifically “Do you smoke?” and if it is a “Yes”, then there are more questions that go on from that and if it is a “No”, then that’s it.’* Midwife [28]

Pressures on professionals’ time could also contribute to this mechanistic approach, particularly the need to complete multiple priority tasks within fixed-length appointments [27, 29, 31].

*‘There’s a lot to be done in the 15 min that we have. We do heights and weights, and we have a lot of paperwork to do along with trying to teach as much as we can… it’s difficult.’* [31]

Whilst these professionals considered they were the right people to be delivering smoking cessation advice, a common barrier, making this difficult to achieve, was the level of staffing, with understaffing a frequent occurrence. [27]. As a result, professionals focused the limited time left on issues raised by women. These centred predominantly on the woman’s health, labour, child development, parenting advice and financial support. This squeezed time for addressing smoking cessation which, in addition, may not be a concern [27, 31].

*‘Whatever you do it always comes down to the labour and that is it…which is fine but giving up smoking isn’t their concern.’* Midwife [27]

## Discussion

To our knowledge, this is the first systematic review of qualitative studies reporting health professionals’ perceptions of the barriers and facilitators they face when addressing smoking cessation with women who are pregnant and in the post-partum period. Using extensive searches from 1990, we identified only eight studies reported in nine papers representing approximately 190 participants. While searching non-English journals may have increased the pool of studies, our review points to an evidence gap, illustrated by the small number of studies available for synthesis.

The small number of studies we had to draw on is a limitation of our review. The studies that were included provide illumination of the barriers and facilitators perceived by health professionals who provide smoking cessation advice and support to women who are pregnant or in the post-partum period. Providing smoking cessation advice during pregnancy is a key part of a health professional’s role and as such the lack of research in this area is surprising particularly in comparison to the substantial body of research with pregnant and post-partum women who smoke [18]. A second potential limitation relates to the methods of qualitative synthesis. These are still being refined [35, 36] and can lack transparency [37]. We therefore used an established methodology for coding and synthesis. In addition, computer software (ATLAS.ti) provided ‘an audit trail’ of the interpretative process and the review was reported in line with the ‘Enhancing Transparency in Reporting the Synthesis of Qualitative Research’ (ENTREQ) guidance [36].

Whilst acknowledging these limitations, our review uncovered recurrent perceptions and experiences among healthcare providers as to the barriers and facilitators they encountered in everyday practice in relation to their work on smoking cessation. The common dimensions related particularly to professionals’ roles and organisational contexts, which were widely seen as shaping barriers and facilitators to supporting smoking cessation. Building on these findings, it is possible to draw some broad interpretations about professional perspectives.

The association between maternal smoking and social disadvantage identified by health professionals as a barrier to addressing and supporting smoking cessation was evidenced by both the quotes presented from health professionals and the authors’ interpretations available in the included studies. Here, professional perceptions of why woman smoke in pregnancy mirrored those of women themselves; a habit deeply entrenched in disadvantaged lives where it provides a source of support, enjoyment and escape [18]. This understanding, together with an acknowledgement that health professionals could not address the social determinants of women’s smoking, heightened professionals’ awareness of the limitations of their role. Perhaps because of this, it was perceived that many women would not or could not quit smoking in pregnancy, and if they did, post-partum relapse was inevitable.

Despite an awareness of this barrier, professionals gave many examples of innovative practice. Here, they drew on their professional knowledge, using experience of ‘what worked’ in the past. Positive and non-judgemental approaches focussed on the woman were seen as the key to successful cessation: encouraging women to take small steps towards quitting, encouraging cutting down as a means to quit and using positive messages around the health of the baby. Where necessary, professionals would adopt a more punitive stance, highlighting the negative effects of smoking on the baby in pregnancy and via second hand smoke after birth. Involving women’s partners in smoking cessation advice was seen to facilitate quitting; however, engaging partners was difficult and, at times, intimidating. Most of these approaches are underpinned by evidence on effective interventions, but some, including advising cutting down, are not. Professionals clearly drew on their own views of what was useful and acceptable to women and partners in addition to any training of knowledge of the evidence that they had.

A major influence on professionals’ approaches to women regarding smoking cessation was the importance attached to their relationship with the woman. A trusting relationship was seen as a prerequisite to fulfilling their responsibilities to the woman and her baby, including around smoking cessation. While potentially facilitating cessation advice and support, the value attached to the relationship could also act as a barrier; professionals were concerned that, unless approached with care and sensitivity, the relationship could be damaged.

Other factors were also identified as potential barriers. This included a lack of knowledge and skills. Of particular note were perceived gaps around effective interventions for women in disadvantaged circumstances and around the prescribing of NRT.

Barriers also included wider organisational constraints. Procedures and time pressures that resulted in ‘tick box’ approaches to smoking were cited as particular barriers. Conversely, clear policies, strong inter-agency links and appropriate investment in woman-focused smoking cessation support, including community-based services, were identified as facilitating smoking cessation.

These broad interpretations provide some pointers for policy and practice. Two inter-linked implications are identified.

Firstly, there is a role for professional education, both pre-qualification training and post-qualification programmes of continuing professional development. It is known that training programs for health professionals which encourage them to ask people if they smoke and offer advice to help them quit, aids both the identification of smokers and increases quit rates [38]. Key within this population however is recognising the centrality of the professional-client/patient relationship, particularly for disadvantaged groups and where continuity of care is limited and services are under strain. This requires professionals having ways of addressing smoking without a perceived risk to their relationship with the woman. These approaches could build directly on approaches that experienced professionals have found helpful and effective in discussing and supporting cessation. Further, as new methods are introduced into routine practice, including the use of carbon monoxide monitoring, concerns about negative impacts on the professional relationship should be recognised and skills provided to minimise these risks.

Secondly, the review points to a widespread professional perception that there is little that healthcare providers can do that is effective in helping women give up smoking in pregnancy, particularly for those in disadvantaged circumstances. Improving the quality and accessibility of evidence on effective healthcare interventions, including evidence on ‘what works’ to support smoking cessation in disadvantaged groups, should therefore be a priority. Equally important is a wider acknowledgement that, while effective in individual cases, support by healthcare providers is unlikely on its own to break the link between social disadvantage and smoking in pregnancy due to the multifaceted nature of disadvantage experienced by many women. Here, our review points to the wisdom and experience of frontline healthcare providers as an important resource for intervention development. Harnessing this untapped resource could help to place the professional’s relationship with the pregnant smoker at the heart of interventions that address the circumstances of smokers’ lives.

## Conclusion

The review comprises a synthesis of eight individual studies reporting on the views and experiences of 190 health professionals/key informants and highlights some of the significant factors associated with health professionals’ role in provision of smoking cessation support for pregnant women. It indicates that there is a manifest need for pre-qualification and continuing professional development across different groups of health professionals involved in promoting smoking cessation. This is underscored by the widespread professional perception that there is little that healthcare providers can do that is effective in helping women give up smoking in pregnancy, particularly for those living in disadvantaged circumstances. Improving the quality and accessibility of evidence on effective healthcare interventions, including evidence on ‘what works’ to support smoking cessation in disadvantaged groups, should therefore be a priority. The review also reveals that health professionals view the professional-client/patient relationship as key to any interactions that take place regarding smoking cessation, and that clinicians may be disinclined to introduce any comments that could be interpreted as judgemental and/or critical, with the potential to undermine the nature of this relationship. Educational programmes will therefore need to take account of this potential barrier to promoting smoking cessation.

## Additional file

Additional file 1: Contains the full search strategy used for the systematic review. (PDF 117 kb)
