# Supplementary material for: Health professionals’ perceptions of the barriers and facilitators to providing smoking cessation advice to women in pregnancy and during the post-partum period: a systematic review of qualitative research
Source: BMC Public Health. 2016 Mar 31;16:290. doi: 10.1186/s12889-016-2961-9 (PMC4815177; doi:10.1186/s12889-016-2961-9)
Supplement: Additional file 1: — Contains the full search strategy used for the systematic review. (PDF 117 kb) [file 12889_2016_2961_MOESM1_ESM.pdf]

Additional File 1: Search strategy for Flemming et al "*Health professionals' perceptions of the barriers and facilitators to providing smoking cessation advice to women in pregnancy and during the post-partum period: a systematic review of qualitative research*". http://dx.doi.org/10.1186/s12889-016-2961-9

CINAHL
Searched via EBSCO interface, 26.02.14
Limiters - Published Date: 19900101-20141231; English Language

#	Query
S75	S74 AND Limiters - Published Date: 19900101-20141231; English Language
S74	S4 AND S11 AND S28 AND S73
S73	S29 OR S30 OR S31 OR S32 OR S33 OR S34 OR S35 OR S36 OR S37 OR S38 OR S39 OR S40 OR S41 OR S42 OR S43 OR S44 OR S45 OR S46 OR S47 OR S48 OR S49 OR S50 OR S51 OR S52 OR S53 OR S54 OR S55 OR S56 OR S57 OR S58 OR S59 OR S60 OR S61 OR S62 OR S63 OR S64 OR S65 OR S66 OR S67 OR S68 OR S69 OR S70 OR S71 OR S72
S72	MH "Patient Education"
S71	MH "Research, Midwifery"
S70	MH "Practice Patterns"
S69	MH "Patient History Taking+"
S68	MH "Professional-Patient Relations"
S67	MH "Professional-Client Relations"
S66	MH "Professional-Family Relations"
S65	MH "Physician-Patient Relations"
S64	MH "Nurse-Patient Relations"
S63	MH "Community Role"
S62	MH "Professional Role"
S61	MH "Physician's Role"
S60	MH "Nursing Role"
S59	MH "Employee Attitudes"
S58	MH "Midwife Attitudes"
S57	MH "Attitude of Health Personnel+"
S56	TI ((health OR "health care" OR healthcare OR medical OR hospital) N2 (personnel OR worker# OR provider# OR employee# OR staff OR professional#)) OR AB ((health OR "health care" OR healthcare OR medical OR hospital) N2 (personnel OR worker# OR provider# OR employee# OR staff OR professional#))
S55	(MH "Pharmacists") OR (MH "Pharmacy Technicians") OR (MH "Pharmacy Service+")
S54	TI (pharmacist# OR pharmacy OR pharmacies) OR AB (pharmacist# OR pharmacy OR pharmacies)
S53	MH "Allied Health Personnel+"
S52	MH "Community Health Workers"
S51	MH "Community Health Nursing"
S50	MH "Home Health Aides"
S49	TI ((home OR health) N2 visit*) OR AB ((home OR health) N2 visit*)
S48	MH "Physicians, Family"
S47	TI (GP# OR "general practitioner" OR "general practitioners") OR AB (GP# OR "general practitioner" OR "general practitioners")
S46	TI (doctor# OR physician# OR clinician#) OR AB (doctor# OR physician# OR clinician#)
S45	TI ((ante-natal OR antenatal) N1 (appointment# OR care OR service#)) OR AB ((ante-natal OR antenatal) N1 (appointment# OR care OR service#))
S44	TI (maternity N1 (care OR service#)) OR AB (maternity N1 (care OR service#))
S43	TI (consultant* N1 (obstetric* OR gyn#ecologic*)) OR AB (consultant* N1 (obstetric* OR gyn#ecologic*))
S42	MH "Obstetrics"
S41	TI (nursing N2 (staff OR personnel OR auxiliar*)) OR AB (nursing N2 (staff OR personnel OR auxiliar*))
S40	TI (nurse#) OR AB (nurse#)
S39	(MH "Obstetric Nursing") OR (MH "OB-GYN Nurse Practitioners")
S38	(MH "Pediatric Nursing") OR (MH "Pediatric Nurse Practitioners")
S37	(MH "Family Nursing") OR (MH "Family Nurse Practitioners")

S36	MH "Maternal-Child Nursing+"
S35	MH "Nurse Midwifery"
S34	MH "Midwives+"
S33	TI (midwi?e*) OR AB (midwi?e*)
S32	TI ((train* OR service* OR support*) N2 cessation) OR AB ((train* OR service* OR support*) N2 cessation)
S31	TI ((train* OR service* OR support*) N2 smoking) OR AB ((train* OR service* OR support*) N2 smoking)
S30	TI ((advisor* OR assist* OR manager* OR professional* OR specialist*) N2 cessation) OR AB ((advisor* OR assist* OR manager* OR professional* OR specialist*) N2 cessation)
S29	TI ((advisor* OR assist* OR manager* OR professional* OR specialist*) N2 smoking) OR AB ((advisor* OR assist* OR manager* OR professional* OR specialist*) N2 smoking)
S28	S12 OR S13 OR S14 OR S15 OR S16 OR S17 OR S18 OR S19 OR S20 OR S21 OR S22 OR S23 OR S24 OR S25 OR S26 OR S27
S27	TI "expectant mother*" OR AB "expectant mother*"
S26	(MH "Expectant Mothers")
S25	TI (pregnant or pregnanc*) OR AB (pregnant or pregnanc*)
S24	(MH "Breast Feeding")
S23	(MH "Postnatal Period+")
S22	(MH "Infant, Newborn")
S21	(MH "Analgesia, Obstetrical")
S20	(MH "Labor Pain")
S19	(MH "Prenatal Care")
S18	(MH "Perinatal Care")
S17	(MH "Prenatal Diagnosis+")
S16	(MH "Fetal Monitoring+")
S15	(MH "Fetus+")
S14	(MH "Maternal Health Services+")
S13	(MH "Pregnancy Complications+")
S12	(MH "Pregnancy+")
S11	S5 OR S6 OR S7 OR S8 OR S9 OR S10
S10	(MH "Tobacco, Smokeless")
S9	(MH "Tobacco")
S8	(MH "Nicotine")
S7	(MH "Smoking Cessation Programs")
S6	(MH "Smoking")
S5	TI ((stop* or quit* or reduc* or give up or giving up) N2 (cigarette* or tobacco or smoking)) OR AB ((stop* or quit* or reduc* or give up or giving up) N2 (cigarette* or tobacco or smoking))
S4	S1 OR S2 OR S3
S3	qualitative
S2	interview* or (MH "Interviews+")
S1	findings

PsycINFO
Searched via EBSCO interface, 25.02.14
Limiters - Publication Year: 1990-2014; English Language

#	Query
S56	S55 AND Limiters - Publication Year: 1990-2014; English
S55	S4 AND S10 AND S22 AND S54
S54	S23 OR S24 OR S25 OR S26 OR S27 OR S28 OR S29 OR S30 OR S31 OR S32 OR S33 OR S34 OR S35 OR S36 OR S37 OR S38 OR S39 OR S40 OR S41 OR S42 OR S43 OR S44 OR S45 OR S46 OR S47 OR S48 OR S49 OR S50 OR S51 OR S52 OR S53
S53	DE "Client Education"
S52	DE "Health Screening"

S51	DE "Professional Identity"
S50	DE "Role Perception"
S49	DE "Health Personnel Attitudes"
S48	DE "Employee Attitudes"
S47	TI ((health OR "health care" OR healthcare OR medical OR hospital) N2 (personnel OR worker# OR provider# OR employee# OR staff OR professional#)) OR AB ((health OR "health care" OR healthcare OR medical OR hospital) N2 (personnel OR worker# OR provider# OR employee# OR staff OR professional#))
S46	DE "Pharmacists"
S45	TI (pharmacist# OR pharmacy OR pharmacies) OR AB (pharmacist# OR pharmacy OR pharmacies)
S44	DE "Allied Health Personnel"
S43	DE "Home Visiting Programs"
S42	DE "Home Care Personnel"
S41	TI ((home OR health) N2 visit*) OR AB ((home OR health) N2 visit*)
S40	DE "General Practitioners"
S39	DE "Family Physicians"
S38	TI (GP# OR "general practitioner" OR "general practitioners") OR AB (GP# OR "general practitioner" OR "general practitioners")
S37	TI (doctor# OR physician# OR clinician#) OR AB (doctor# OR physician# OR clinician#)
S36	TI ((ante-natal OR antenatal) N1 (appointment# OR care OR service#)) OR AB ((ante-natal OR antenatal) N1 (appointment# OR care OR service#))
S35	TI (maternity N1 (care OR service#)) OR AB (maternity N1 (care OR service#))
S34	TI (consultant* N1 (obstetric* OR gyn#ecologic*)) OR AB (consultant* N1 (obstetric* OR gyn#ecologic*))
S33	DE "Obstetrics" OR DE "Obstetricians"
S32	TI (nursing N2 (staff OR personnel OR auxiliar*)) OR AB (nursing N2 (staff OR personnel OR auxiliar*))
S31	TI (nurse#) OR AB (nurse#)
S30	DE "Public Health Service Nurses"
S29	DE "Nurses"
S28	DE "Midwifery"
S27	TI (midwi?e*) OR AB (midwi?e*)
S26	TI ((train* OR service* OR support*) N2 cessation) OR AB ((train* OR service* OR support*) N2 cessation)
S25	TI ((train* OR service* OR support*) N2 smoking) OR AB ((train* OR service* OR support*) N2 smoking)
S24	TI ((advisor* OR assist* OR manager* OR professional* OR specialist*) N2 cessation) OR AB ((advisor* OR assist* OR manager* OR professional* OR specialist*) N2 cessation)
S23	TI ((advisor* OR assist* OR manager* OR professional* OR specialist*) N2 smoking) OR AB ((advisor* OR assist* OR manager* OR professional* OR specialist*) N2 smoking)
S22	S11 OR S12 OR S13 OR S14 OR S15 OR S16 OR S17 OR S18 OR S19 OR S20 OR S21
S21	TI "expectant mother*" OR AB "expectant mother*"
S20	TI (pregnant or pregnanc*) OR AB (pregnant or pregnanc*)
S19	DE "Breast Feeding"
S18	DE "Postnatal Period"
S17	DE "Prenatal Care" OR DE "Childbirth Training"
S16	DE "Perinatal Period"
S15	DE "Prenatal Diagnosis"
S14	DE "Prenatal Exposure"
S13	DE "Fetus"

S12	DE "Obstetrical Complications"
S11	DE "Pregnancy" OR DE "Adolescent Pregnancy"
S10	S5 OR S6 OR S7 OR S8 OR S9
S9	DE "Passive smoking"
S8	DE "Smokeless Tobacco"
S7	DE "Nicotine"
S6	DE "Smoking Cessation"
S5	DE "Tobacco Smoking"
S4	S1 OR S2 OR S3
S3	qualitative
S2	interview* or (DE "Interviews") or (DE "Interviewing")
S1	findings

Medline
Searched via EBSCO interface, 26.02.14
Limiters - Date of Publication: 19900101-20141231; English Language

#	Query
S67	S66 AND Limiters - Date of Publication: 19900101-20141231; English Language
S66	S4 AND S14 AND S26 AND S65
S65	S27 OR S28 OR S29 OR S30 OR S31 OR S32 OR S33 OR S34 OR S35 OR S36 OR S37 OR S38 OR S39 OR S40 OR S41 OR S42 OR S43 OR S44 OR S45 OR S46 OR S47 OR S48 OR S49 OR S50 OR S51 OR S52 OR S53 OR S54 OR S55 OR S56 OR S57 OR S58 OR S59 OR S60 OR S61 OR S62 OR S63 OR S64
S64	MH "Patient Education as Topic"
S63	MH "Nursing Methodology Research"
S62	MH "Medical History Taking"
S61	MH "Physician's Practice Patterns"
S60	MH "Physician-Patient Relations"
S59	MH "Nurse-Patient Relations"
S58	MH "Physician's Role"
S57	MH "Nurse's Role"
S56	MH "Attitude of Health Personnel"
S55	TI ((health OR "health care" OR healthcare OR medical OR hospital) N2 (personnel OR worker# OR provider# OR employee# OR staff OR professional#)) OR AB ((health OR "health care" OR healthcare OR medical OR hospital) N2 (personnel OR worker# OR provider# OR employee# OR staff OR professional#))
S54	(MH "Pharmacists") OR (MH "Pharmacists' Aides") OR (MH "Pharmaceutical Services+")
S53	TI (pharmacist# OR pharmacy OR pharmacies) OR AB (pharmacist# OR pharmacy OR pharmacies)
S52	MH "Allied Health Personnel"
S51	MH "Community Health Workers"
S50	(MH "Community Health Nursing+") OR (MH "Nurses, Community Health")
S49	MH "Home Health Aides"
S48	TI ((home OR health) N2 visit*) OR AB ((home OR health) N2 visit*)
S47	MH "Physicians, Family"
S46	MH "General Practitioners"

S45	TI (GP# OR "general practitioner" OR "general practitioners") OR AB (GP# OR "general practitioner" OR "general practitioners")
S44	TI (doctor# OR physician# OR clinician#) OR AB (doctor# OR physician# OR clinician#)
S43	TI ((ante-natal OR antenatal) N1 (appointment# OR care OR service#)) OR AB ((ante-natal OR antenatal) N1 (appointment# OR care OR service#))
S42	TI (maternity N1 (care OR service#)) OR AB (maternity N1 (care OR service#))
S41	TI (consultant* N1 (obstetric* OR gyn#ecologic*)) OR AB (consultant* N1 (obstetric* OR gyn#ecologic*))
S40	MH "Obstetrics"
S39	TI (nursing N2 (staff OR personnel OR auxiliar*)) OR AB (nursing N2 (staff OR personnel OR auxiliar*))
S38	TI (nurse#) OR AB (nurse#)
S37	MH "Obstetric Nursing"
S36	(MH "Public Health Nursing") OR (MH "Nurses, Public Health")
S35	(MH "Pediatric Nursing") OR (MH "Pediatric Nurse Practitioners")
S34	(MH "Family Nursing") OR (MH "Family Nurse Practitioners")
S33	MH "Maternal-Child Nursing"
S32	(MH "Midwifery") OR (MH "Nurse Midwives")
S31	TI (midwi?e*) OR AB (midwi?e*)
S30	TI ((train* OR service* OR support*) N2 cessation) OR AB ((train* OR service* OR support*) N2 cessation)
S29	TI ((train* OR service* OR support*) N2 smoking) OR AB ((train* OR service* OR support*) N2 smoking)
S28	TI ((advisor* OR assist* OR manager* OR professional* OR specialist*) N2 cessation) OR AB ((advisor* OR assist* OR manager* OR professional* OR specialist*) N2 cessation)
S27	TI ((advisor* OR assist* OR manager* OR professional* OR specialist*) N2 smoking) OR AB ((advisor* OR assist* OR manager* OR professional* OR specialist*) N2 smoking)
S26	S15 OR S16 OR S17 OR S18 OR S19 OR S20 OR S21 OR S22 OR S23 OR S24 OR S25
S25	TI "expectant mother*" OR AB "expectant mother*"
S24	MH "Pregnant Women"
S23	TI (pregnant or pregnanc*) OR AB (pregnant or pregnanc*)
S22	MH "Maternal Health Services+"
S21	MH "Breast Feeding"
S20	MH "Postpartum Period+"
S19	MH "Prenatal Care"
S18	MH "Perinatal Care"
S17	MH "Prenatal Diagnosis+"
S16	MH "Infant, Newborn"
S15	MH "Pregnancy+"
S14	S5 OR S6 OR S7 OR S8 OR S9 OR S10 OR S11 OR S12 OR S13
S13	MH "Tobacco Smoke Pollution"
S12	MH "Tobacco, Smokeless"
S11	MH "Nicotine"
S10	MH "Smoking Cessation"
S9	MH "Tobacco Use Cessation"
S8	MH "Tobacco Use Disorder"
S7	TI ((stop* or quit* or reduc* or give up or giving up) N2 (cigarette* or tobacco or smoking)) OR AB ((stop* or quit* or reduc* or give up or giving up) N2 (cigarette* or tobacco or smoking))
S6	MH "Tobacco"

S5	MH "Smoking"
S4	S1 OR S2 OR S3
S3	qualitative
S2	interview* or MH "Interview"
S1	findings

Social Sciences Citation Index (SSCI)
Searched via Web of Knowledge interface, 25.02.14
Databases=SSCI; Timespan=1990-2014; Language=(English)

#21	(#19 AND #3 AND #2 AND #1) *AND* LANGUAGE: *(English)*
#20	(#19 AND #3 AND #2 AND #1)
#19	#18 OR #17 OR #16 OR #15 OR #14 OR #13 OR #12 OR #11 OR #10 OR #9 OR #8 OR #7 OR #6 OR #5 OR #4
#18	TS=((health OR "health care" OR healthcare OR medical OR hospital) NEAR/2 (personnel OR worker$ OR provider$ OR employee$ OR staff OR professional$))
#17	TS=(pharmacist$ OR pharmacy OR pharmacies)
#16	TS=((home OR health) NEAR/2 visit*)
#15	TS=(GP$ OR "general practitioner" OR "general practitioners")
#14	TS=(doctor$ OR physician$ OR clinician$)
#13	TS=((ante-natal OR antenatal) NEAR/1 (appointment$ OR care OR service$))
#12	TS=(maternity NEAR/1 (care OR service$))
#11	TS=(consultant* NEAR/1 (obstetric* OR gyn$ecologic*))
#10	TS=(nursing NEAR/2 (staff OR personnel OR auxiliar*))
#9	TS=(nurse$)
#8	TS=(midwi?e*)
#7	TS=((train* OR service* OR support*) NEAR/2 cessation)
#6	TS=((train* OR service* OR support*) NEAR/2 smoking)
#5	TS=((advisor* OR assist* OR manager* OR professional* OR specialist*) NEAR/2 cessation)
#4	TS=((advisor* OR assist* OR manager* OR professional* OR specialist*) NEAR/2 smoking)
#3	TS=(pregnant or pregnanc* or "expectant mother*")
#2	TS=(cigarette* or tobacco or smok* or nicotine)
#1	TS=(qualitativ* or finding* or interview*)

The Economic and Social Research Council (ESRC)
Searched via http://www.esrc.ac.uk/search/advanced-search.aspx, 26.02.14
(The ESRC website's advanced search engine is configured to use the Porter Stemming plugin which reduces search words down to their base word.)

	All of these words:	AND	Any of these words:
1.	qualitative pregnant		cigarette tobacco smoker smoking nicotine
2.	interview pregnant		cigarette tobacco smoker smoking nicotine
3.	finding pregnant		cigarette tobacco smoker smoking nicotine
4.	qualitative pregnancy		cigarette tobacco smoker smoking nicotine
5.	interview pregnancy		cigarette tobacco smoker smoking nicotine
6.	finding pregnancy		cigarette tobacco smoker smoking nicotine
7.	qualitative expectant		cigarette tobacco smoker smoking nicotine
8.	interview expectant		cigarette tobacco smoker smoking nicotine
9.	finding expectant		cigarette tobacco smoker smoking nicotine
10.	qualitative *midwife*		cigarette tobacco smoker smoking nicotine
11.	interview *midwife*		cigarette tobacco smoker smoking nicotine
12.	finding *midwife*		cigarette tobacco smoker smoking nicotine
13.	qualitative *midwives*		cigarette tobacco smoker smoking nicotine
14.	interview *midwives*		cigarette tobacco smoker smoking nicotine

15.	finding *midwives*	cigarette tobacco smoker smoking nicotine
16.	qualitative *nurse*	cigarette tobacco smoker smoking nicotine
17.	interview *nurse*	cigarette tobacco smoker smoking nicotine
18.	finding *nurse*	cigarette tobacco smoker smoking nicotine
19.	qualitative *professional*	cigarette tobacco smoker smoking nicotine
20.	interview *professional*	cigarette tobacco smoker smoking nicotine
21.	finding *professional*	cigarette tobacco smoker smoking nicotine
22.	qualitative *advisor*	cigarette tobacco smoker smoking nicotine
23.	interview *advisor*	cigarette tobacco smoker smoking nicotine
24.	finding *advisor*	cigarette tobacco smoker smoking nicotine
25.	qualitative *GP*	cigarette tobacco smoker smoking nicotine
26.	interview *GP*	cigarette tobacco smoker smoking nicotine
27.	finding *GP*	cigarette tobacco smoker smoking nicotine

PubMed (including Medline)
Searched via NCBI interface, 28.02.14
Limited to Ahead of Print Citations and articles published in the last 3 months.

Search	Query
#7	#5 OR #6
#6	#4 AND (2013/12:2014 [edat] OR 2013/11:2014 [crdt] OR 2013/12:2014[dp])
#5	#4 AND pubstatusaheadofprint
#4	#1 AND #2 AND #3
#3	"Pregnant Women"[mesh] OR "Breast Feeding"[mesh] OR "Postpartum Period"[mesh] OR "Infant, Newborn"[mesh] OR "Obstetric Surgical Procedures"[mesh] OR "Analgesia, Obstetrical"[mesh] OR "Labor Pain"[mesh] OR "Prenatal Care"[mesh] OR "Perinatal Care"[mesh] OR "Prenatal Diagnosis"[mesh] OR "Fetal Therapies"[mesh] OR "Fetal Monitoring"[mesh] OR "Fetus"[mesh] OR "Maternal Health Services"[mesh] OR "Pregnancy Complications"[mesh] OR "Pregnancy"[mesh] OR pregnant[tiab] OR pregnanc*[tiab] OR "expectant mother*"[tiab]
#2	"Tobacco Smoke Pollution"[mesh] OR "Tobacco, Smokeless"[mesh] OR "Tobacco"[mesh] OR "Nicotine"[mesh] OR "Tobacco Use Disorder"[mesh] OR "Tobacco Use Cessation"[mesh] OR "Smoking Cessation"[mesh] OR "Smoking"[mesh] OR ((stop*[tiab] OR quit*[tiab] OR reduce*[tiab] OR reduct*[tiab] OR "give up" [tiab] OR "giving up" [tiab]) AND (cigarette*[tiab] OR tobacco[tiab] OR smoking[tiab]))
#1	findings OR qualitative OR interview* OR "Interviews as Topic"[mesh] OR "Interview, Psychological"[mesh]

Google Scholar
Searched via http://scholar.google.co.uk, 28.02.14
Limited to English language pages only and 2014 to capture 'ahead of print' but 'published online' articles excluding patents; cookies deleted between searches.

All of these words:	This exact phrase:	Any of these words:
qualitative pregnant	published online	cigarette tobacco smoker smoking nicotine
interview pregnancy	published online	cigarette tobacco smoker smoking nicotine
qualitative "expectant"	published online	cigarette tobacco smoker smoking nicotine
interview "expectant"	published online	cigarette tobacco smoker smoking nicotine
